# Validity, Reliability and Responsiveness of the Malay Shoulder Pain and Disability Index (M-SPADI) for Patients with Shoulder Pain

**DOI:** 10.5704/MOJ.2303.019

**Published:** 2023-03

**Authors:** S Abdul-Karim, MS Abdul-Hamid, CA Ho, JCY Ling

**Affiliations:** 1 Department of Sports Medicine, Universiti Malaya, Kuala Lumpur, Malaysia; 2 Department of Biomedical Engineering, Universiti Malaya, Kuala Lumpur, Malaysia; 3 Department of Sports Medicine, Tengku Ampuan Rahimah Hospital, Klang, Malaysia

**Keywords:** measurement properties, SPADI, reliability, responsiveness, validity

## Abstract

**Introduction:**

The purpose of this study is to determine the validity, reliability, and responsiveness of the Malay Shoulder Pain and Disability Index (M-SPADI) in Malay speakers suffering from shoulder pain.

**Materials and methods:**

The M-SPADI, the Numerical Rating Scale (NRS), and measurements of shoulder active range of motion (AROM) were completed by 140 patients with shoulder pain (68 with rotator cuff pathology and 72 with other shoulder pathology). Thirty-four patients were retested for test-retest reliability with M-SPADI after an average of 9.2 days. M-SPADI was performed on twenty-one individuals three months after completing treatment for rotator cuff disorders to assess response.

**Results:**

The results of exploratory factor analysis revealed a bidimensional structure for M-SPADI. M-SPADI disability score was significantly greater in patients with rotator cuff pathologies (median = 31.87, IQR 82.50) than in patients with other shoulder pathologies (median = 20.00, IQR 23.84). In multi-group factor analysis, measurement invariance revealed no significant difference between the two groups (p>0.05). There was a significant positive correlation between M-SPADI and NRS (Pain = 0.86, Disability = 0.75, Total = 0.82, p=0.005), and a significant negative correlation between M-SPADI and shoulder AROM (Pain = -0.34 to -0.67, Disability =-0.44 to -0.73, Total =0.43 to -0.72, p=0.005). M-SPADI had a high degree of internal consistency (Cronbach's 0.92 for pain and 0.95 for disability). Test-retest reliability was moderate to excellent (ICC Pain = 0.84, ICC Disability = 0.78, ICC Total = 0.81, p=0.001), and the smallest detectable change ranges (Pain = 8.74, Disability = 3.21, Total = 3.83) were less than the minimal detectable change ranges (Pain = 21.57, Disability = 6.82, Total = 8.79). The area under the receiver operating characteristic curve (AUC) for M-SPADI was greater than 0.90 (Pain = 0.99, Disability = 0.94, Total = 0.96).

**Conclusion:**

The M-SPADI has established construct validity, internal consistency, test-retest reliability, and responsiveness. The M-SPADI is a reliable and valid instrument for evaluating shoulder pain among Malay-speaking individuals. In addition, the M-SPADI disability subscale may be useful for monitoring functional score changes in patients with rotator cuff pathology.

## Introduction

Patient-reported outcomes measures (PROM) are structured, validated questionnaires that patients complete to evaluate their perceived level of impairment, disability, and health-related quality of life^1^. It is frequently used in research as a primary or secondary outcome measure^2^. Numerous PROMs exist to quantify pain and physical function or activity. Three types of PROM commonly used for the shoulder are upper extremity region-specific (e.g. Disability of Arm, Shoulder, and Hand [DASH]), shoulder-specific (e.g. American Shoulder and Elbow Surgeon Score [ASES] and Shoulder Pain and Disability Index [SPADI])and disease-specific (e.g. Western Ontario Rotator Cuff Index [WORC] and Oxford Shoulder Instability Score [OSIS])^3^. The evaluation of pain and physical function were identified as key outcome domains in most studies^4^. The Shoulder Pain and Disability Index (SPADI) is one of the most frequently used PROMs among patients with shoulder pain^5^.

The SPADI was developed in 1991 and since then has been validated in groups of patients with rotator cuff and subacromial pathology, calcific tendinitis, adhesive capsulitis, and non-specific shoulder pain6. It was also responsive to change in various clinical settings, such as treatment for adhesive capsulitis, subacromial impingement, and shoulder arthroplasty^7,8^. The SPADI is self-administered and takes between 2 and 5 minutes to complete^9^. It has been translated and validated into many languages, including Danish^10^, Dutch^11^, Spanish^12^, Telugu^13^, Chinese^14^, and Slovene^15^.

The Malay SPADI (M-SPADI) had previously been validated in the general population, that include patients with non-specified shoulder pain and patients without shoulder pain^16^. The previous study only considered a limited set of psychometric properties including content validity, convergent validity, known-group validity (shoulder pain and no shoulder pain groups), hypothesis testing, and test-retest reliability. The Consensus-based Standards for the Selection of Health Measurement Instruments (COSMIN) guidelines proposed ten criteria that can be used to determine whether a study meets the standards for good methodologic quality: content validity, internal structure (including structural validity and internal consistency), reliability, measurement error, hypotheses testing, cross-cultural validity, criterion validity, and responsiveness^17^.

Hence, this study evaluated the translated Malay SPADI (M-SPADI) in the context of the COSMIN guidelines to provide additional methodologic quality assessment, particularly for patients with shoulder pain.

## Materials and Methods

The research was conducted using a prospective cohort validation design that was approved by the Universiti Malaya Medical Centre (UMMC), Medical Research Ethics Committee (MECID No. 201977-7623). The study was conducted in accordance with the Helsinki Declaration, and prior to participation, all patients provided written informed consent. Patients were recruited from an outpatient clinic in a university hospital between June 2020 and May 2021. Patients had to be over the age of 18, able to speak in Malay and presented with shoulder pain to be considered for the study. Exclusion criteria included injuries to the neck, elbow, wrist, or hand, unwillingness to provide informed consent, or a diagnosis of a mental disorder.

A physician from the research team completed the screening, which included a review of the medical record for radiological results and clinical assessments. Following screening, patients who agreed to participate must provide written informed consent. Patients sociodemographic information including age, gender, occupation, level of education, and duration of shoulder discomfort we gathered using a standardised clinical research form. Participants were then required to complete the M-SPADI in Appendix 1 and verbal Numerical Rating Scale (NRS). The patients' active range of motion (AROM) of the shoulder was also evaluated in all functional planes for the shoulder. This includes flexion, extension, abduction, adduction and internal and external rotation.

From the group of patients mentioned above, patients who had no invasive procedures or new treatments in the weeks following their enrolment in the study were asked to complete the M-SPADI again seven days later to assess test-retest reliability. Patients who received treatment for shoulder pain, on the other hand, were approached three months later to repeat the M-SPADI to assess responsiveness.

In this study two important tools used were the M-SPADI questionnaire and the Numerical Rating Scale (NRS). The M-SPADI is a self-administered questionnaire developed to assess shoulder pain and impairment in adults, and it was translated from the original SPADI by Roach *et al*^6^. It consists of thirteen items that evaluate two distinct domains. The first five questions assess patients' pain, while the following eight are on functional disability. The M-SPADI scoring includes the Total M-SPADI, the combined score for both domains, since few questions assess both domain^16^. The M-SPADI questionnaire uses an 11-point numerical scale on which the individual rates their level of pain or disability on a scale of 0 to 10 using the anchors “no pain to worst pain imaginable” or “no difficulty to so difficult it requires assistance”^18^. The sum of the subscale item scores was divided by the maximum possible subscale score and multiplied by 100 to obtain a score out of 100. A score was considered invalid if more than two items were missing^6^. The scores range from 0 to 100, with no cut-off point to indicate severity, as they were intended to measure current conditions and change over time^5^.

The NRS is a frequently used tool to assess pain intensity. Typically, 0 denotes "no pain," while 10 denotes "the worst pain imaginable"^19^. It allows for a more detailed comparison of pain levels than the VAS, which allows for an infinite number of possible responses. Numerous studies have established strong correlations between the NRS and other tools for assessing pain. Moreover, the NRS can be used easily with a good compliance rate^20,21^.

The validity study sample size was estimated based on the recommendations for the participant-to-item ratio of a minimum of 5:1 (five participants to one item)^22^. In addition, Mokkink *et al* suggested that the sample size should be more than 100 to be qualified for analysis^23^. Thus, in this study, 140 patients with shoulder pain were recruited.

For the M-SPADI test-retest reliability, the estimated sample size was calculated using the computer software downloaded from http://wnarifin.github.io^24^. Intercorrelation coefficient (ICC) with an expected ICC of 0.95, precision of 0.5, confidence level of 95%, number of repetitions per subject of 2, and a drop-out rate of 10%, the minimum sample size needed was 20.

All statistical analyses of the M-SPADI measurement properties were conducted using IBM SPSS Statistics for Windows, version 27 (IBM Corp., Armonk, NY, USA) and smart-PLS version 3.2.8. The significance level for all statistical tests was set at 0.05, and the normality of all data was determined using Q-Q plots and the Kolmogorov-Smirnov test. Non-parametric tests were performed when data was not normally distributed.

Construct validity was evaluated as convergent, discriminant, and known-group validity. In addition, we include measurement of invariance analysis. Convergent validity assesses the degree of agreement between multiple indicators of the same construct. To establish convergent validity, the indicator's factor loading must be greater than 0.7, composite reliability (CR) or Cronbach’s more than 0.6, and the average variance extracted (AVE) greater than 0.525. The degree to which two constructs are empirically distinct is termed discriminant validity. It also quantifies the degree to which the overlapping structures differ. To determine discriminant validity, the Fornell and Larcker criterion was used. According to the criteria, the square root of the AVE of each construct should be greater than the correlations with other latent constructs^26^. Additionally, the group validity was assessed by comparing the rank means of M-SPADI scores among patients with rotator cuff pathology with other causes of shoulder pain. The U test was used to determine the validity. We hypothesised that patients with rotator cuff pathology would have higher M-SPADI disability scores than patients with other shoulder pain and that the difference would be statistically significant (p<0.05).

Measurement invariance refers to the degree to which respondents from different groups with the same latent trait level respond similarly to a given subscale^23^. Measurement invariance was calculated using a multiple group factor analysis. Positive results were expected when there were no significant differences between the group factors^27^. We hypothesised that there would be no significant differences in the factors between patients with rotator cuff pathology and those with other shoulder pathologies.

For criterion validity, Spearman's correlation was used to determine the correlation between M-SPADI and the NRS, as well as the correlation between M-SPADI and shoulder AROM. The relationships were classified as weak (Spearman's rho 0.30–0.50), or strong (Spearman's rho >0.70)^28^. We hypothesised a high positive correlation between the M-SPADI and the NRS. Furthermore, M-SPADI would have a significant negative correlation with shoulder AROM.

The reliability tests consist of internal consistency, test-retest reliability, and measurement error. The M-SPADI internal consistency was assessed using Cronbach's alpha (α) and average inter-item correlation for each M-SPADI subscale. Cronbach's α≥0.70 for each subscale was considered good^22^. The average inter-item correlation is a criterion for determining whether individual questions on a test or questionnaire produce consistent, appropriate results; items measuring the same general construct or concept are compared for similar scores. The ideal average inter-item correlation range is 0.15 to 0.5029. Correlation <0.15 suggested items are not well correlated and do not accurately measure the same construct or idea. Correlation greater than 0.50 indicates items are so close to each other that they are redundant^30^.

For test-retest reliability, based on the average measurement model, the Cronbach’s α must be more than 0.70 for all subscales to be considered reliable. The interclass correlation coefficient (ICC), calculated with a 95% confidence interval, must be more than 0.70 to be considered satisfactory^31^. The interval between repeated assessments was determined to be between seven and fourteen days to prevent recall and ensure there was no clinical change^32^.

Absolute reliability was determined by assessing the measurement error. The Standard Error of Measurement (SEM) and the Smallest Detectable Change (SDC) were analysed. The standard error of the mean (SEM) denotes the standard deviation (SD) of repeated measurements in a single patient, and N = sample size (SEM = SD√N)^33^. SDC is the smallest change sufficient to establish that the observed change is real and not attributable to measurement error (SDC = SEM x √N x1.96)^34^. A measure of variability associated with the SEM is the minimal detectable change (MDC), which is the smallest detectable change that can be considered above the measurement error with a given level of 95% confidence. The SDC should be less than the MDC for a good measurement error test^17^.

For responsiveness, the receiver operating curve (ROC) method was used to examine the ability of the M-SPADI to detect treatment response over time. The patients were classified as “improved” if the NRS score was <3 and “unchanged” if the NRS >435. The assessment was conducted three months after individuals with rotator cuff tendinopathy had treatment with regenerative therapy and exercise. In ROC curve analysis, sensitivity and specificity are plotted at several cut-off points, and the area under the curve (AUC) can be estimated. AUCs greater than 0.70 are thought to be adequate for demonstrating that outcome measures can distinguish between improved and unimproved patients^36^.

## Results

One hundred forty patients were enrolled in the validity study. Sixty-eight participants were diagnosed with rotator cuff pathologies and 72 with other shoulder conditions. The Q-Q plots and Kolmogorov-Smirnov tests revealed the M-SPADI scores, NRS scores, and shoulder AROM were not normally distributed. Analysis based on a non-parametric test found no significant differences in the sociodemographic characteristics between the two groups, as shown in Table I.

**Table I:** Patients’ sociodemographic characteristic.

Parameters	Patients with rotator cuff pathology (n=68)	Patients with other shoulder pathology (n=72)	p-value
Age (years), Median (IQR)	51.58 (56.75)	54.12 (69.19)	0.344*
Gender, N (%)			
Male	34 (50%)	38 (53%)	0.866**
Female	34 (50%)	34 (47%)	
Occupation, N (%)			
Employed	43 (63%)	45 (62%)	0.964**
Retired	6 (9%)	7 (10%)	
Homemaker	15 (22%)	17 (24%)	
Other	4 (6%)	3 (4%)	
Education, N (%)			
Primary education	2 (3%)	1 (1%)	0.786**
Secondary education	19 (28%)	22 (31%)	
Tertiary education	68 (69%)	72 (68%)	
Duration of pain in weeks, Median (IQR)	28 (1036.00)	24 (519.70)	0.351*

Abbreviation - IQR: Inter-quartile range, N: number

Notes: * Mann-Whitney test, ** chi-square. p- value is considered statistically significance if <0.05

Convergent validity was demonstrated by an AVE greater than 0.5 for both items, composite reliability (CR) greater than 0.7, factor loading greater than 0.6, and Cronbach's alpha greater than 0.7. Table II illustrates a good fit to convergence for all of the constructs. Thus, all of the items are measuring what they were designed to measure.

**Table II:** Construct validity determine by convergent and discriminant validity.

Construct	Convergent			Discriminant (Fornell-Lacker)	
	Cronbach’s α	CR	AVE	M-SPADI Pain	M-SPADI Disability
M-SPADI Pain	0.923	0.942	0.766	0.875	-
M-SPADI Disability	0.984	0.957	0.736	0.834*	0.858

Abbreviation - CR: composite reliability, AVE: extracted average variance (AVE), M-SPADI: Malay Shoulder Pain and Disability Index

Notes: * The number represented the correlation of a shared construct less than the correlation of each construct

Discriminant validity was assessed based on the Fornell-Larcker criterion. The criteria showed the correlation of M-SPADI Pain with M-SPADI Disability were less than the square root of the individual AVE, thus successfully establishing the discriminant validity of the two factors (pain and disability) (Table II).

The M-SPADI Disability was significantly higher in patients with rotator cuff pathology (median = 31.87, IQR 82.50) compared to other shoulder pathologies (median = 20.00, IQR 23.84) (U = 1933.5, z = -2.15, p = 0.032). Similarly, the M-SPADI total was significantly higher in patients with rotator cuff pathology (median = 36.92, IQR = 79.23) than in other shoulder conditions (median = 24.62, IQR = 85.38), U = 1951.5, z =-2.07, p = 0.038 (Table III).

**Table III:** 

	Rotator cuff Pathology (n=68)	Other shoulder pain (n=72)			
Score	Mean Rank		U	z	p
M-SPADI Pain	77.10	64.26	1999.00	-1.873	0.061
M-SPADI Disability	78.07	63.35	1933.50	-2.146	0.032*
M-SPADI Total	77.80	63.60	1951.50	-2.070	0.038*
Multi-group analysis	Path coefficient				p
M-SPADI Pain and M-SPADI Disability	0.019				0.703
M-SPADI Pain and Total M-SPADI	0.018				0.508
M-SPADI Disability and M-SPADI Total	0.022				0.425

Note: * p- value <0.05.

A multi-group analysis using confirmatory factor analysis revealed that patients with rotator cuff pathologies and other shoulder pathologies had no statistically significant differences between group factors for the M-SPADI Pain, M-SPADI Disability, and M-SPADI Total, p>0.05 (Table III).

Criterion validity indicates a substantial positive correlation between M-SPADI Pain, M-SPADI Disability, and M-SPADI Total and NRS. Moreover, a strong inverse correlation was observed between M-SPADI Pain, Disability, Total and shoulder AROM. All AROM associations were from moderate to high, except for shoulder extension (-0.36 to -0.44), which ranged from weak to moderate (Table IV).

**Table IV:** Correlation between M-SPADI with NRS and M-SPADI with active range of motion (AROM) for Criterion validity.

Scale	M-SPADI Pain	M-SPADI Disability	M-SPADI Total
NRS	.858*	.747*	.815*
AROM			
Forward flexion	-.668*	-.730*	-.722*
Abduction	-.636*	-.731*	-.713*
Extension	-.358*	-.444*	-.434*
Internal rotation 90°	-.540*	-.676*	-.645*
External rotation 90°	-.511*	-.608*	-.593*

Abbreviation - NRS: Numeric Pain Rating Scale, AROM: Active range of motion

Notes: * Significance correlations, p<0.05

For internal consistency, calculations of the average inter-item correlation between the five items on the M-SPADI Pain questionnaire reveal that all items correlate. The average range of inter-item correlation was 0.26 indicating that inter-item correlation were fairly consistent. Similarly, the average inter-item correlation between the eight M-SPADI Disability items indicates that all items correlate with one another with the average inter-item correlation range of 0.265, indicating good consistency (Table V). The two-part questionnaire, which was completed by 140 patients, revealed that the M-SPADI pain subscale (Cronbach α = 0.92), and the disability subscale, (Cronbach α = 0.948), had excellent internal consistency.

**Table V:** Internal consistency of the M-SPADI Pain and M-SPADI Disability measured with Cronbach’s α and Average Inter-Item Correlation.

Scales	Cronbach's α	Average Inter-item Correlation	
		Range	Average
M-SPADI Pain	0.923	0.259	0.805
M-SPADI Disability	0.948	0.265	0.764

Abbreviation - M-SPADI: Malay Shoulder Pain and Disability Index

Notes: Cronbach's α>0.70 and range of average inter-item correlation between 0.15 to 0.50 was considered good22.

Thirty-four patients completed the test-retest with a mean time of 9.20± 3.80 days between the first and second tests. The Cronbach’s α was more than 0.70 in all subscales. The ICC results of the M-SPADI pain subscale, disability subscale, and total scores (ICC Pain =0.84, ICC Disability =0.78, ICC Total =0.81, p<0.001) revealed good to excellent test-retest reliability (Table VI). The measurement error associated with the M-SPADI was shown in Table VI. In M-SPADI Pain (SDC=8.74) and M-SPADI Disability (SDC=3.21), the SDC was lower than the MDC. The Bland-Altmann analysis was omitted because the values for the difference between test and retest scores were not normally distributed^37^.

**Table VI:** Test-retest reliability of the M-SPADI assessed by Cronbach’s α, Interclass correlation coefficient and measurement error.

Scales	Cronbach's α	Interclass Correlation Coefficient		Measurement Error		
		ICC	95% CI	SEM	MDC	SDC
M-SPADI Pain	0.83	0.84	0.67, 0.92	7.78	21.57	8.74
M-SPADI Disability	0.78	0.78	0.56, 0.89	2.46	6.82	3.21
M-SPADI Total	0.81	0.81	0.62, 0.91	3.17	8.79	3.83

Abbreviation – ICC: Interclass correlation coefficient, SEM: Standard Error of Measurement, M-SPADI: Malay Shoulder Pain and Disability Index, MDC: Minimal detectable change, SDC: Smallest detectable change

**Fig. 1:**
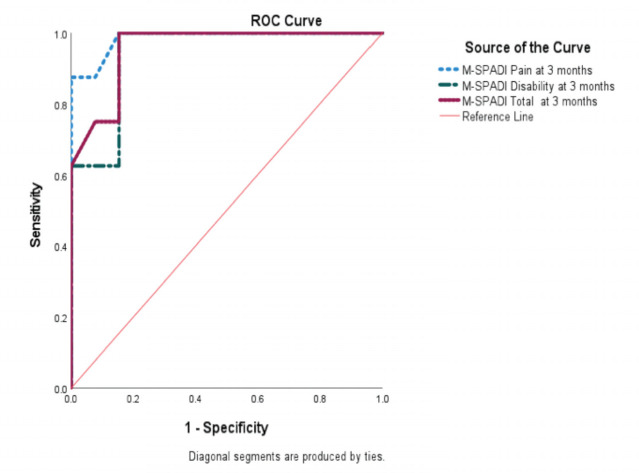
Responsiveness: Area under the curve for M-SPADI Pain, M-SPADI Disability and M-SPADI Total at three months after treatment.

Twenty-one patients completed the responsiveness test at three months’ post-treatment with regenerative therapy and shoulder rotator cuff strengthening exercise. The AUC for MSPADI pain, disability, and total SPADI was more than 0.90, indicating that the M-SPADI is highly responsive (Table VII and Fig. 1).

**Table VII:** Responsiveness of patients with rotator cuff pathology after treatment with regenerative therapy and exercise.

Scale	AUC	95% Confidence interval	Sensitivity	Specificity
M-SPADI Pain	0.99	0.95-1.00	1.00	0.23
M-SPADI Disability	0.94	0.85-1.00	1.00	0.15
SPADI Total	0.96	0.88-1.00	1.00	0.23

Abbreviation - AUC: Area under curve, M-SPADI: Malay Shoulder Pain and Disability Index

## Discussion

In this study the translated M-SPADI was evaluated using the COSMIN guidelines. Aside from COSMIN, there are other instruments available to evaluate the measurement characteristics of patient-reported outcome measures including "Evaluating Measures of Patient-Reported Outcomes (EMPRO)," "Quality Criteria for Measurement Properties" and “Scientific Advisory Committee Medical Outcomes Trust (SACMOT)"^38^. Among these, COSMIN was reported to be the most comprehensive and frequently used to assess the psychometric properties of self-reported measures^39^. As a thorough and rigorous assessment method developed by an international team of experts by Delphi agreement, the COSMIN guideline offers multiple strengths^17^. COSMIN has the following advantages: the instructions for conducting and analysing patients' reported outcome measures were thoroughly explained and are readily available online. In addition, it offers guidelines for Classical Test Theory (CTT) and Item Response Theory (IRT)38. Although some concerns were raised on the complexity of COSMIN as it involves multiple steps of evaluations, COSMIN provides a robust description of interpretability, burden, and alternative modes of administration compared to the rest^39^.

The M-SPADI revealed high validity and reliability, as well as adequate responsiveness, in patients with shoulder pain. According to the structural validity, the M-SPADI has bidimensional structure; the MSPADI pain and disability^16^. The questionnaire's bi-dimensionality were also identified by the original SPADI^6^, the Slovene SPADI^15^, and the Chinese SPADI^14^ and in two large study participants using the Rasch model^10,40,41^.

The M-SPADI known-group validity found that patients with shoulder pain had considerably greater pain and disability scores than those without shoulder pain^16^. In this study, we found two main clusters of shoulder pain, which were: rotator cuff related pathologies and other shoulder pathologies which include bursitis, acromioclavicular joint disease, adhesive capsulitis, instability and majority was non-specific shoulder pain. Thus, a known group validity between the two groups were performed. Patients with rotator cuff disease had significantly higher M-SPADI Disability and M-SPADI Total scores than patients with other shoulder pathologies. A similar finding was reported by Tran *et al*, which found that patients with rotator cuff pathology had the highest SPADI score compared to other pathologies. They suggested poor response to treatment among patient with rotator cuff pathology as were the cause of this observation^42^. Similarly, the current study found patients with rotator cuff pathology have longer duration of the condition which could be associated with higher M-SPADI score. Moreover, rotator cuff pathology is often a chronic condition that is challenging to treat^43,44^ and often associated with pain and functional limitation^45,46^. In contrast patients with other shoulder pathologies such as subacromial bursitis and adhesive capsulitis usually respond well to treatment (e.g. corticosteroid injection and physiotherapy modalities)^47^. Our findings may reflect that M-SPADI could be utilised to assess rotator cuff pathology comparable with rotator cuff-specific shoulder questionnaires such as the Western Ontario Rotator Cuff (WORC) Index, since patients with rotator cuff pathology showed significantly higher disability scores compared to other shoulder pathologies. Furthermore, there were studies that reported good validity and reliability of SPADI for patients with rotator cuff disease^40,48^. There was no other translated SPADI that could be compare for the known group validity to the present study.

The cross-cultural validity or measurement invariance was defined broadly in the COSMIN guidelines as “the degree to which the performance of items on a translated or culturally adapted instrument is a sufficient reflection of the performance of the items in the original version or of a different ethnic group, gender, or patient population”^17^. There were various ways assessing the measurement invariance for the SPADI. Thoomes-de Graaf *et al*^11^ compared the Dutch SPADI in patients with a high initial pain score level of >7 who were absent from work with patients with a lower initial pain score level of less than 7 who were not absent from work. In his study, patients with a high initial pain score with work absence had a significantly higher mean SPADI score than those with a low initial pain score. Other study compared pain on the dominant side versus the non-dominant side found significantly higher SPADI pain scores on the dominant side than the non-dominant side^40^. In this study, the measurement invariance was assessed by comparing patients with rotator cuff pathology and with other types of shoulder pain. According to the multi-group factor analysis, there was no significant difference in measurement invariance between the two groups. This measurement invariance is important to evaluate if patients who have different traits (rotator cuff problems and other shoulder problems) understand the questionnaire in the same way^17^.

In this study, M-SPADI was found to be strongly correlated with NRS and negatively correlated with active range of motion in terms of criterion validity. This finding is comparable with the M-SPADI Spanish^12^ and China^14^ which demonstrated a significant correlation of M-SPADI with NRS. Spearman's correlation validated the hypothesis that M-SPADI subscales and overall scores were negatively correlated with shoulder AROM. SPADI has previously been associated with NRS and AROM in the Original SPADI^6^, Telugu SPADI^13^, and Tamil SPADI^49^.

All of the items in M-SPADI Pain and M-SPADI Disability had good internal consistency, with a Cronbach's alpha greater than 0.90. As a result, neither the pain subscale nor the disability subscale questions should be removed, nor should additional questions be added. Three items were removed from the Spanish SPADI to achieve the best fit model for confirmatory factor analysis^12^. Our findings with Cronbach’s alpha of more than 0.90 consistent with previous research using the SPADI questionnaire in other shoulder diseases^48,49^.

The test-retest reliability for M-SPADI was rated as good to excellent and was superior to that of the original SPADI, which had moderate test-retest reliability^6^. The difference could be due to smaller sample size of 37 patients in the latter study and the patients with non-specific shoulder pain was recruited. A recent systematic review reports test-retest reliability of SPADI ranges from ICC = 0.85-0.92, similar to this study^5^. The test-retest is important because it determines the consistency of the patients responses to the questionnaire over time^31^.

Only a few studies examined the SPADI's measurement error^50-53^. The term "measurement error" refers to the systematic and random variation in a patient's score that cannot be attributed to true changes in the construct being measured^23^. The SEM in this study was between 2.46 and 7.78, which is consistent with previous studies that reported SEM between 2.1 and 8.950,51. Previous studies reported the MDC values from 12.2 to 23.1 (50-52). The MDCs for M-SPADI Disability (6.82) and M-SPADI Total (8.79) were low in this study, indicating that the majority of patients had similar pathology (rotator cuff). According to the COSMIN guidelines, the MDC should ideally be greater than the SDC^23^.

A limited number of studies assessed the responsiveness of the SPADI overtime or after treatment^32^. Responsiveness is defined as the ability of a PROM to detect change over time in the construct being measured^23^. The AUC in this study was comparable with other studies, which ranged from 0.80 to 0.9250,51,54.

In this study, the AUC for patients with rotator cuff tendinopathy treated with regenerative therapy injection combined with rotator cuff exercise program, was greater than 0.90 at three months. AUC greater than 0.90 indicates that the M-SPADI could discriminate between patients who responded to treatment and those who did not respond to treatment as assessed by NRS. According to Kc *et al*, the overall quality of the SPADI responsiveness study was moderate, with inconsistent findings^56^. In contrast our study demonstrated excellent responsiveness to intervention. However, the comparison was difficult to perform because the majority of studies did not include information on the treatment received by the patients^6,49,55,56^.

There are several limitations that need to be addressed in this study. First, we did not verify whether patients remained stable during the 7-day period between the test and the retest, which could have influenced the measurement error. However, the seven-day period between assessments used is commonly used in previous studies^50,55^. Second, previous studies compared the SPADI construct validity with other validated questionnaires^48,51,54^. However, given the absence of other validated Malay shoulder questionnaire comparison could not be made. For the purpose of determining responsiveness, we only analysed patients diagnosed with rotator cuff tendinopathy, as this is the most common condition seen in our setting. The patients were treated with regenerative therapy and exercise for chronic pain. The strength of this study is that it supplements the results of the previous study by including a more comprehensive psychometric properties assessment which include the criterion validity, measurement invariance and responsiveness^16^. Additionally, this study demonstrates previously undetermined known-group validity and measurement invariance, which compares patients with and without rotator cuff problems.

Validated health-related patient-reported outcome measures (PROMs) is essential in promoting patient-centred care as it provides the clinician with abundant information to assist decision-making in the various aspects of patient care including diagnostic process, treatment selection, monitoring treatment progress, and facilitating effective communication with patients. There are at least fifty PROMs available on shoulder function, with the Shoulder Pain and Disability Index (SPADI) being one of the most frequently referenced and studied^3,57^. The SPADI questionnaire is brief, simple to use, easy to comprehend, and had substantial evidence for validity, reliability, and responsiveness56. Moreover, the SPADI has been translated into at least fifteen other languages. The M-SPADI, is the first shoulder-specific questionnaire translated into Malay that is valid and reliable, and could be utilised in clinical and research settings to assess pain and disability among Malay-speaking individuals with shoulder pain. The findings of this study are comparable to those of other international studies employing the SPADI questionnaire and would be useful for future large cross-cultural and cross-regional study.

## Conclusion

The M-SPADI is a bi-dimensional instrument with moderate/good/excellent validity, reliability, and responsiveness for research and monitoring patient response to treatment. M-SPADI may be used to monitor the progression of disability and functions in patients with rotator cuff pathology, including their post-treatment response and progression.
